# Sustained Clinical Improvement in a Subset of Patients With Progressive Multiple Sclerosis Treated With Epstein–Barr Virus-Specific T Cell Therapy

**DOI:** 10.3389/fneur.2021.652811

**Published:** 2021-03-15

**Authors:** Zara A. Ioannides, Peter A. Csurhes, Nanette L. Douglas, Gem Mackenroth, Andrew Swayne, Kate M. Thompson, Tracey J. Hopkins, Kerryn A. Green, Stefan Blum, Kaye D. Hooper, Kerstin H. Wyssusek, Alan Coulthard, Michael P. Pender

**Affiliations:** ^1^Faculty of Medicine, The University of Queensland, Brisbane, QLD, Australia; ^2^Department of Neurology, Royal Brisbane and Women's Hospital, Herston, QLD, Australia; ^3^The University of Queensland Centre for Clinical Research, Herston, QLD, Australia; ^4^Mater Centre for Neurosciences, Mater Hospital, Brisbane, QLD, Australia; ^5^Neurology Department, Princess Alexandra Hospital, Woolloongabba, QLD, Australia; ^6^Department of Psychology, Royal Brisbane and Women's Hospital, Herston, QLD, Australia; ^7^School of Psychology, The University of Queensland, Brisbane, QLD, Australia; ^8^Internal Medicine Day Treatment Unit, Royal Brisbane and Women's Hospital, Herston, QLD, Australia; ^9^Department of Anesthesia and Perioperative Medicine, Royal Brisbane and Women's Hospital, Herston, QLD, Australia; ^10^Department of Medical Imaging, Royal Brisbane and Women's Hospital, Herston, QLD, Australia

**Keywords:** B cell, CD8^+^ T cell, disease-modifying therapy, Epstein-Barr virus, progressive multiple sclerosis, T cell therapy

## Abstract

**Background:** Increasing evidence indicates a role for Epstein–Barr virus (EBV) in the pathogenesis of multiple sclerosis (MS). EBV-infected autoreactive B cells might accumulate in the central nervous system because of defective cytotoxic CD8^+^ T cell immunity. We have previously reported results of a phase I clinical trial of autologous EBV-specific T cell therapy in MS 6 months after treatment.

**Objective:** To investigate longer-term outcomes in MS patients who received autologous EBV-specific T cell therapy.

**Methods:** We assessed participants 2 and 3 years after completion of T cell therapy.

**Results:** We collected data from all 10 treated participants at year 2 and from 9 participants at year 3. No serious treatment-related adverse events were observed. Four participants had at least some sustained clinical improvement at year 2, including reduced fatigue in three participants, and reduced Expanded Disability Status Scale score in two participants. Three participants experienced a sustained improvement in at least some symptoms at year 3. More sustained improvement was associated with higher EBV-specific CD8^+^ T cell reactivity in the administered T cell product.

**Conclusion:** Autologous EBV-specific T cell therapy is well-tolerated, and some degree of clinical improvement can be sustained for up to 3 years after treatment.

## Introduction

Multiple sclerosis (MS) is a chronic inflammatory demyelinating disease of the central nervous system (CNS) characterized by progressive neurological disability. Although the primary cause of MS is unknown, genetic and environmental factors contribute to the risk of developing MS. A mounting body of evidence indicates that infection with Epstein–Barr virus (EBV) has an important role in the pathogenesis of MS (1, 2). In particular, the universality of EBV infection in people with MS (3) including those with early MS (4), and the presence of high titres of anti-EBV antibodies in the sera of patients prior to the onset of MS (5–8) and during the course of MS (9) indicate that infection with EBV has a critical role in the development of MS.

In our 2003 hypothesis, we postulated that defective elimination of EBV-infected B cells by cytotoxic CD8^+^ T cells might predispose to the development of MS by enabling the accumulation of EBV-infected autoreactive B cells in CNS (10). Our hypothesis is supported by observations of an accumulation of EBV-infected B cells and plasma cells in the brain in MS (11–14), although some studies have not found EBV infection in the brain (15, 16). A key prediction from the hypothesis is that EBV-specific T cell therapy should kill EBV-infected autoreactive B cells in the CNS, thereby halting disease progression and resulting in clinical improvement (10, 17).

AdE1-LMPpoly is an adenoviral vector encoding CD8^+^ T cell epitopes from EBV nuclear antigen 1 (EBNA1), latent membrane protein 1 (LMP1), and LMP2A which has been used to stimulate and expand EBV-specific CD8^+^ T cells in people with nasopharyngeal carcinoma, where the carcinoma cells express these EBV proteins (18, 19). Given that EBV-infected B cells in the brain in MS express the same three EBV proteins (11, 20), we postulated that T cells expanded with AdE1-LMPpoly might kill EBV-infected B cells in the CNS and thus halt disease progression and lead to clinical improvement in people with MS. In 2013, we first treated an MS patient with EBV-specific T cell therapy (21). The patient was a 42-year-old man with secondary progressive MS (SPMS); following autologous EBV-specific T cell therapy there was sustained clinical improvement and decreased cerebrospinal fluid (CSF) immunoglobulin G (IgG) production for 3.5 years after T cell therapy.

Following this, in 2015, we commenced an investigator-led open-label phase 1 clinical trial of autologous EBV-specific T cell therapy in patients with progressive MS (22). There were no treatment-related serious adverse effects and seven of the ten patients who received T cell therapy experienced clinical improvement. Of these, three patients showed an improvement in the Expanded Disability Status Scale (EDSS) score (23). Clinical improvement after T cell therapy was related to the level of EBV-specific CD8^+^ T cell reactivity in the T cell product. All six participants receiving a T cell product with strong EBV reactivity (>5% of CD8^+^ T cells reacting to EBV) showed clinical improvement whereas only one of the four participants receiving a T cell product with weak EBV reactivity (<5% of CD8^+^ T cell reacting to EBV) showed improvement (*p* = 0.033, Fisher's exact test) (22).

The objective of the current study was to investigate the longer-term clinical course of progressive MS in patients treated with autologous EBV-specific T cell therapy by performing comprehensive assessments at 2 and 3 years after treatment.

## Materials and Methods

### Study Approval

Our original phase I clinical trial of EBV-specific T cell therapy is registered with the Australian New Zealand Clinical Trials Registry (ACTRN12615000422527). The current 3-year follow-up study was approved by the Royal Brisbane and Women's Hospital Human Research Ethics Committee and The University of Queensland Medical Research Ethics Committee. All participants provided written informed consent.

### Subjects

The study population consisted of the patients with progressive forms of MS, as defined by the 2010 Revised McDonald Criteria (24), who received autologous EBV-specific T cell therapy in our previously published phase I clinical trial (22). These were 5 patients with primary progressive MS (PPMS, 2 males, and 3 females) and 5 patients with SPMS (2 males and 3 females) who were between the ages of 42 and 61 at the time of receiving T cell therapy (Table 1). Patients were assessed at 2 years and at 3 years after completion of T cell therapy.

**Table 1:** Time of clinical assessments, intercurrent medical conditions, and adverse events during the observation period.

**Participant code (age in years^a^, sex, form of MS)**	**Time of year 2 assessment (months^b^)**	**Time of year 3 assessment (months^b^)**	**Intercurrent medical condition and adverse events related to a study procedure (associated treatment)**.
1 (49, F, SPMS)	24	36	Urinary tract infection at 23 months (methenamine). Cataract of left eye at 36 months (laser-assisted cataract surgery).
2 (54, F, SPMS)	24	Participant did not attend	Subacute deterioration in chronic suicidality due to change in social circumstances at 12 months (supportive treatment).
3 (61, M, SPMS)	24	35	Atrial fibrillation at 8 months (rivaroxiban and metoprolol). Mechanical fall resulting in a right orbital floor fracture at 25 months (supportive treatment). Localized stage 0 melanoma and intra-epidermal carcinoma excised at 31 months. Diverticulosis and polyps of the sigmoid colon detected at 35 months (resection of polyps).
4 (49, F, PPMS)	24	36	Hypothyroidism at 33 months (thyroxine).
5 (60, F, SPMS)	24	36	Osteoporosis at 24 months (zoledronic acid). Back pain and bruising at LP site performed at year 2 and 3 (supportive treatment).
6 (53, M, PPMS)	23	35	Back pain at LP site performed at year 2 (supportive treatment).
8 (42, F, PPMS)	Participant did not attend^c^	Participant did not attend^c^	Urinary tract infection and deep vein thrombosis of the lower limb at 17 months (rivaroxaban and antibiotics)
9 (46, M, SPMS)	24	40	Bruise at site of blood collection performed at year 2 (supportive treatment). Intestinal pseudo-obstruction at 18 months (aperients and enemas)
12 (60, M, PPMS)	22	36	NA
13 (55, F, PPMS)	22	36	NA

a *age at time of receiving EBV-specific T cell therapy*.

b *months after completion of EBV-specific T cell therapy*.

c *with the participant's consent, clinical information was obtained retrospectively from electronic medical records up to 36 months after T cell therapy*.

*PPMS, primary progressive multiple sclerosis; SPMS, secondary progressive multiple sclerosis; LP, lumbar puncture; NA, No intercurrent medical condition or adverse event related to study procedure was reported by the participant or observed. All references to time points refer to the time following the completion of EBV-specific T cell therapy. Participant 2 did not attend for the year 3 assessment. Participants 7, 10, and 11 did not receive T cell therapy in the Phase 1 clinical trial and were not included in this follow-up paper*.

### Clinical Assessments

At each assessment neurological symptoms were assessed in a face-to-face interview with a neurologist. At each visit we obtained a detailed medical history, including the temporal profile of any changes in neurological symptoms; performed a clinical examination, including a detailed neurological examination; and assessed the EDSS score (24). To assess fatigue, we administered the self-reported Fatigue Severity Scale (25). To assess quality of life (QOL) a single question from the MS QOL Instrument was used; this question asks “Overall, how would you rate your own quality of life?” on a scale from 0 (worst—as bad or worse than being dead) to 10 (best), as used by Cosio et al. (26).

To evaluate cognitive function, a clinical neuropsychologist administered a comprehensive neuropsychological test battery at 2 and 3 years following T cell therapy. The Minimal Assessment of Cognitive Function in Multiple Sclerosis (MACFIMS) formed the basis of the comprehensive cognitive assessment and is a validated battery for the assessment of cognitive function in patients with MS (27). The MACFIMS was supplemented with a measure of reaction time, the Computerized Test of Information Processing, and a screen of depressive symptoms, the Beck Depression Inventory (BDI)—Fast Screen for Medical Patients (28). To minimize the impact of practice effects due to repeated assessment, alternate forms of three measures (the Brief Visual Memory Test, the California Verbal Learning Test—Second Edition, and the Delis-Kaplan Executive Function System Sorting Test) were administered in an alternating fashion according to 10 computer-generated randomization schedules established prior to participant recruitment. Pairwise comparisons of change over time were conducted for each of the 15 tests used in the comprehensive neuropsychological test battery. Scores measured at 2 and 3 years were compared to baseline. Paired *t*-tests or Wilcoxon matched-pairs signed rank test were used according to data distribution as assessed by the Kolmogorov-Smirnov test. After application of statistical corrections for multiple comparisons a *p*-value threshold for significance was set at *p* < 0.0033.

As a mood disorder could affect cognition, in addition to administering the BDI, we also screened patients for depression by asking two questions used by Mohr et al. (29), namely, (a) “During the past 2 weeks, have you often been bothered by feeling down, depressed, or hopeless?” and (b) “During the past 2 weeks, have you often been bothered by little interest or pleasure in doing things.”

At years 2 and 3, brain and spinal cord MRI acquired at 3 Tesla before and after intravenous injection of gadolinium-containing contrast material (including 3-dimensional T1 and 3-dimensional T2 fluid-attenuated inversion recovery sequences for assessment of enhancing lesions and T2 lesions) were performed. Previously published MRI brain scan data from baseline and 6 months following T cell therapy was used to assess the presence of new T2 lesions compared to the prior MRI scans. MRI scans were also assessed for the number of gadolinium-enhancing lesions. Lumbar puncture for CSF analysis of intrathecal IgG production with matched serum analysis was performed at years 2 and 3.

## Results

### Subjects

Nine of the 10 patients who received EBV-specific T cell therapy in the original phase I trial consented to participate in the current follow-up study. Participant 8 was unable to participate due to social circumstances; however, she provided consent for us to access her Queensland Health electronic medical record for up to 3 years after she received T cell therapy and to include relevant data in study reports and/or publication. Participant 2 did not consent to the year 3 assessment. Lumbar puncture was contraindicated in participant 3 at years 2 and 3 because of anti-coagulation. Owing to social circumstances, participant 3 was unable to attend for the MRI scans and neuropsychological assessment at year 3. Participants 7, 10 and 11 from the original phase I clinical trial did not receive T cell therapy and were therefore not included in this follow-up study.

### Intercurrent Medical Conditions, Adverse Events, and New MS Therapy

No treatment-related serious adverse events were observed during the study. Intercurrent medical conditions and adverse events related to a study procedure during the follow-up period are presented in Table 1. Participant 1 commenced ocrelizumab at 33 months after T cell therapy because of an MS relapse (Table 2). Participant 13 commenced ocrelizumab at 34 months after T cell therapy because of a deterioration in walking ability (Table 2).

**Table 2:** Clinical course of MS following EBV-specific T cell therapy.

**Participant code**	**EBV-specific reactivity of CD8^**+**^** ** T cells (%)^a^^,^^b^**	**Clinical course at 6 months^a^**	**Clinical course at 2 years**	**Clinical course at 3 years**
1	0.4	Reduced fatigue. Improved ability to garden, paint, and perform daily activities. Improved balance, with Romberg's sign changing from positive to negative.	Improved ability to perform daily activities was sustained until 10 months. Improvements in fatigue and balance were sustained until 13 months. Romberg's sign was positive at 24 months.	Walking ability and pain symptoms unchanged since baseline. Loss of sensation in the right V2–3 dermatomes, and a gadolinium-enhancing lesion in the right pons on MRI brain scan at 30 months. Treated with intravenous methylprednisolone. Ocrelizumab was commenced at 33 months.
2	0.72	No change from baseline.	Increased leg spasticity at 11 months treated with intravenous methylprednisolone.	Information not available
3	22.25	Improved energy levels and productivity. Improved endurance in swimming and gardening. Improvement in power of left hip and knee flexion but deterioration in left ankle plantarflexion.	Improvements in energy levels, productivity, endurance, and power of left knee flexion sustained. Deterioration in the power of left hip flexion, ankle dorsiflexion, and toe dorsiflexion between month 6 and year 2. Pregabalin commenced for neuropathic pain of the lower limbs at 24 months.	Improvements in energy levels, productivity and endurance sustained. Deterioration in the power of left hip extension and toe dorsiflexion between year 2 and 3.
4	14.16	Reduced fatigue. Better able to converse and concentrate on conversation. Resolution of vertigo. Reduction in diplopia. Increased manual dexterity with utensils. Reduced lower limb spasms. Resolution of symptomatic lower limb clonus. Reduced urinary urgency and 75% reduction in nocturia. Visual acuity improved from 6/9 (20/30) bilaterally to 6/6 (20/20) bilaterally. Color vision (Ishihara plates) improved bilaterally, from recognition of 12 plates to 20 of the total 21 plates for the right eye and from recognition of 15 plates to 19 plates for the left eye.	Reduction in fatigue sustained at 24 months. The resolution of vertigo, reduction in diplopia, reduced lower limb spasms, resolution of symptomatic lower limb clonus, and reduction in urinary urgency and nocturia were sustained at year 2. At 10 months neuropathic lower limb pain resolved and all pain medications were ceased. Visual acuity returned to baseline of 6/9 bilaterally. Color vision (Ishihara plates) improvement from baseline was sustained bilaterally (recognition of 19 of the total 21 plates bilaterally). Left hand function reduced at year 2 due to worsening ataxia.	Fatigue increased. The resolution of vertigo, reduction in diplopia, reduced lower limb spasms, resolution of symptomatic lower limb clonus, and of neuropathic pain were sustained at year 3. Visual acuity was the same as baseline (6/9 bilaterally). Color vision (Ishihara plates) improvement from baseline was sustained bilaterally (recognition of 18 of the total 21 plates bilaterally) Further reduction in left hand function compared to year 2 due to reduction in finger strength and worsening ataxia.
5	45.45	Reduced fatigue. Increased productivity in all activities. Improved manual dexterity—insertion of earrings for first time in years. Improved walking ability from 100 to 1,500 m with wheeled walker and to 100 m with unilateral assistance. Eighty percentage of reduction in nocturia, and improved sleep. Near complete resolution of lower limb spasticity, normalization of lower limb power and knee jerks for first time in 16 years, transient normalization of plantar reflexes from extensor to flexor, normalization of coordination in upper and lower limbs, and resolution of impaired light touch and vibration sense in lower limbs. Improvement in EDSS score from 6.5 to 6.0.	Reduction in fatigue sustained. Improvements in productivity, manual dexterity, and walking ability were sustained. Continued improvement in walking ability to 190 m with unilateral assistance. Sustained improvements in nocturia and sleep. Additional improvements in daytime urinary and fecal urgency. Able to consume a meal and drink in the morning without daytime incontinence for the first time since 6 months prior to receiving T cell therapy. Additional improvement in ability to complete work tasks including better memory for numbers and names. Additional improvement in ability to stand from sitting; no longer required a raised toilet seat.	Fatigue increased but remained lower than baseline level. Improvements in productivity, manual dexterity, sleep, nocturia, and urinary and fecal urgency sustained. Sustained improvement in ability to stand from sitting. Sustained improvement in lower limb spasticity and normalization of distal lower limb power. Sustained normalization of coordination in lower limbs and only subtle incoordination of the left upper limb. Sustained resolution of impaired light touch and vibration sense in lower limbs. EDSS score returned to baseline score of 6.5 because of inability to walk without bilateral assistance. Able to walk 1,000 m with bilateral assistance. Sustained improvement in QOL, 8/10 from 7/10 at baseline.
			Sustained improvement in lower limb spasticity. Distal lower limb power normalized from 2–3/5 at baseline. Sustained normalization of coordination in lower limbs and only subtle incoordination of the left upper limb. Sustained resolution of impaired light touch and vibration sense in lower limbs. EDSS score improvement maintained at 6.0. Sustained improvement in QOL score (9/10 from 7/10 at baseline).	
6	0.34	No change from baseline.	Worsening of walking ability. Deterioration in EDSS score from 6.0 at baseline and 6 months to 7.0. Deterioration in visual acuity of the right eye from 6/12 at baseline to 6/30. Color vision (Ishihara plates) of the right eye deteriorated from recognition of 17 plates at baseline to 14 of the total 21 plates.	Worsening of upper limb ataxia and walking ability. EDSS score remained at 7.0. Visual acuity of the right eye 6/24. Color vision (Ishihara plates) of the right eye deteriorated from recognition of 17 plates at baseline to 12 of the total 21 plates.
8	2.87	Initial improvement in concentration, mental clarity (with lifting of mental fog), and productivity. At week 27 there was an increase in right lower limb pain, spasms, and weakness, with EDSS score increasing from 6.5 at week 21 to 7.0 at week 27 and decreased mood related to social circumstances.	Deterioration in walking ability following urinary tract infection and deep vein thrombosis. EDSS score 7.5 compared to 6.0 at baseline.	Increased leg spasticity and pain. EDSS score 8.0.
9^c^	6.34	Reduced fatigue. Improved cognition and word-finding. Increased productivity. Increased voluntary movements in toes and at left ankle.	Fatigue level lower than baseline but higher than at 6 months. Improved cognition and word-finding sustained. Voluntary movements in toes and left ankle reduced to below baseline level at 7 months. Left hand function deteriorated owing to increasing weakness and ataxia at 10 months. Color vision (Ishihara plates) of the left eye deteriorated from recognition of 21 plates at baseline to 16 of the total 21 plates. EDSS deteriorated from 8.0 at baseline to 8.5 at year 2 due to increased upper limb ataxia.	Fatigue level lower than baseline and 6 months. Improved cognition and word-finding sustained. Color vision (Ishihara plates) of the left eye stable with recognition of 16 of the total 21 plates.
12	29.85	Reduced fatigue. Improved concentration and mental clarity (lifting of mental fog). Improved speech and ability to follow complex conversation. Increased productivity. Sixty percentage of reduction in nocturia. Increased walking distance from 200 to 500 m without aid or rest. Improvement in EDSS score from 5.0 to 3.5.	Reduction in fatigue and nocturia and improvement in walking distance to 500 m without aid or rest sustained until 12 months after T cell therapy. Improvement in concentration and mental clarity sustained until 18 months after T cell therapy. Walking started to deteriorate 12 months after T cell therapy; distance of 100 m with unilateral support and EDSS score of 6.0 at 24 months.	Walking distance 400 m with unilateral support and EDSS score of 6.0.
13	7.90	Improved sleep quality. Improved mood. Improved handwriting. Increased walking distance from 200 to 440 m without aid or rest. Romberg's sign changed from positive to negative. Improvement in EDSS score from 5.0 to 4.5.	Improvement in mood and handwriting sustained until 7 months after T cell therapy. Improved sleep quality sustained at 24 months. Walking distance of 377 m without aid or rest. Romberg's sign remained negative and EDSS score improvement to 4.5 sustained at 24 months.	Ocrelizumab was commenced at 34 months owing to deterioration in walking distance at 32 months (77 m with bilateral assistance). Improved sleep quality sustained at 36 months. Romberg's sign reverted to positive and EDSS score deteriorated to 6.5.

*The clinical course of MS was determined from a face-to-face interview with a neurologist and a neurological examination at year 2 and 3. Where possible, the time point of clinical change relative to the time of completion of T cell therapy was determined from the participant history and/or collateral information from the treating neurologist who assessed the participant between study assessments*.

a *previously reported by Pender et al. (22)*.

b *percentage of CD8^+^ T cells in the T cell product reacting to EBV*.

c *treated with EBV-specific T cell therapy 4 years prior to treatment in the phase I clinical trial (21)*.

*FSS, Fatigue Severity Scale; EDSS, Expanded Disability Status Scale. All references to time points refer to the time following the administration of EBV-specific T cell therapy. Participant 2 was unable to attend for the year 3 assessment owing to social circumstances. Participants 7, 10, and 11 did not receive T cell therapy in the phase 1 clinical trial and were not included in this follow-up paper*.

### Clinical Outcomes Following T Cell Therapy

*The year* 2 *assessment*

**Figure 1:**
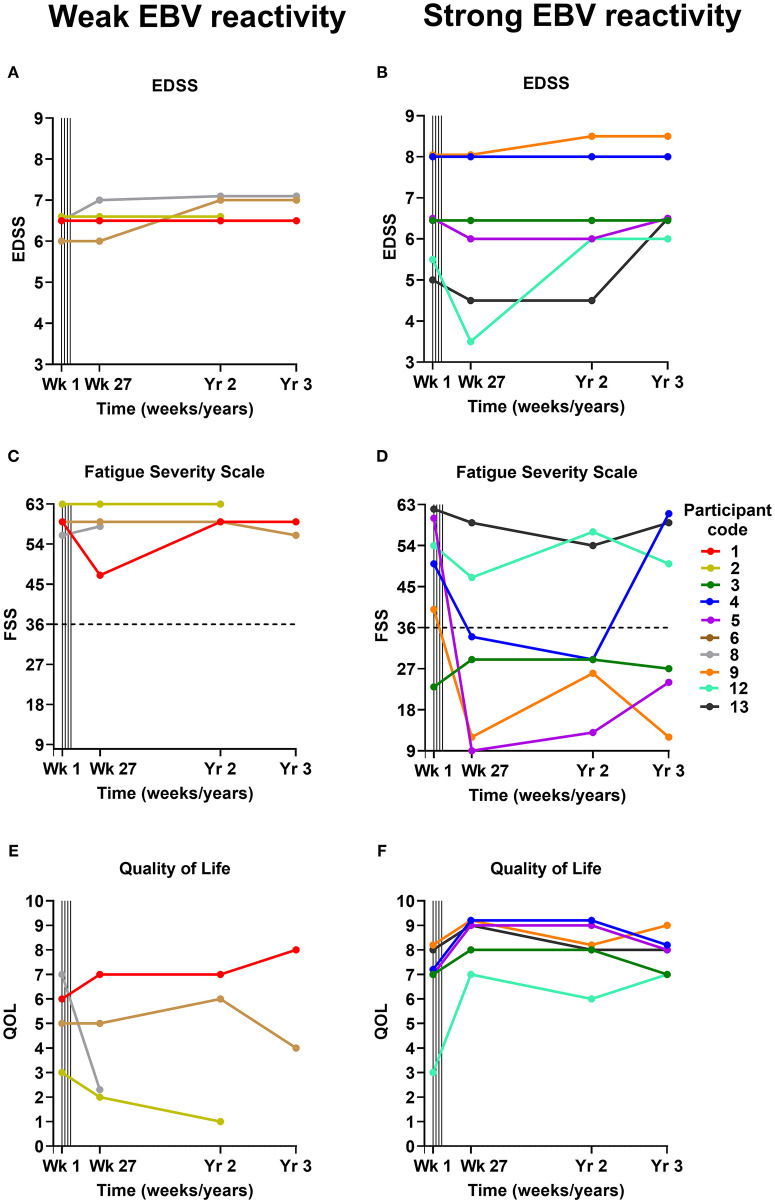
EDSS **(A,B)**, Fatigue Severity Scale **(C,D)**, and Quality of Life **(E,F)** scores before and after EBV-specific T cell therapy. The participants are grouped according to whether they received a T cell product with weak EBV reactivity (<5% of CD8^+^ T cells reacting to EBV) **(A,C,E)** or strong EBV reactivity (>5% of CD8^+^ T cells reacting to EBV) **(B,D,F)**. For the Fatigue Severity Scale **(C,D)** a total score of 36 (indicated by dotted horizontal lines) or more suggests that a person is suffering from fatigue; the maximum score is 63 and the minimum score is 9. Vertical lines indicate successive T cell infusions of 5 × 10^6^, 1 × 10^7^, 1.5 × 10^7^, and 2 × 10^7^ cells at 2-week intervals from week 1 to 7. EDSS, Expanded Disability Status Scale; FSS, Fatigue Severity Scale; QOL, Quality of Life; EBV, Epstein–Barr virus; Wk, week; Yr, year.

Four of the 7 participants who experienced clinical improvement at the 6-month assessment (published previously) (22) were found to have some degree of sustained improvement at the 2-year assessment (participants 3, 4, 5, and 13) (Table 2; Figures 1A-F). Interestingly, the T cell therapy received by all of these 4 participants had strong EBV reactivity [>5% of CD8^+^ T cells reacting to EBV, (22) as presented in Table 2]. In participants 4, 5 and 13 there was a reduction in the Fatigue Severity Scale score at year 2 compared to baseline (Figure 1D). The improvement in QOL score that had been observed at 6 months was sustained at year 2 in participants 3, 4, and 5, but not in participant 13 (Figure 1F). In participant 3, despite the sustained improvements in energy levels, productivity, endurance and power of left knee flexion, lower limb neuropathic pain developed and there was deterioration in the power of other left lower limb movements at year 2. In participant 4, despite the sustained improvement in many symptoms, there was deterioration in left hand function due to worsening ataxia at year 2. In participants 5 and 13 the clinical improvements were accompanied by a sustained improvement in EDSS score at year 2 compared to baseline (Table 2 and Figure 1B). Indeed, participant 5 showed further improvements in memory, ability to complete work tasks, daytime urinary and fecal urgency, and walking ability at year 2, compared to month 6 after T cell therapy. The T cell therapy received by participants 5 and 13 not only had strong EBV reactivity but also reactivity to all 3 EBV proteins, namely EBNA1, LMP1, and LMP2A (22). A deterioration in MS symptoms and/or neurological examination findings between the 6-month and the 2-year assessments was observed in 6 participants (participants 1, 2, 6, 8, 9, and 12), of whom 4 (participants 1, 2, 6, and 8) received T cell therapy with weak EBV reactivity (<5% of CD8^+^ T cells reacting to EBV) (Table 2). The duration of clinical improvement in participants 1, 9, and 12, who experienced clinical improvement at the 6-month assessment that was not sustained at the 2-year assessment, was 13 months in participant 1, 10 months in participant 9, and 12 months in participant 12 (Table 2). The EDSS in participant 9 deteriorated from 8.0 at baseline and month 6 to 8.5 at year 2 owing to worsening of upper limb ataxia; however, the improvements in cognition and fatigue which were noted by the participant at month 6 were sustained at year 2 and at year 3. In this participant the duration of sustained clinical improvement (10 months) after administration of the T cell product in this clinical trial (6.34% of CD8^+^ T cells reacted to EBV) was substantially less than after the participant's first course of EBV-specific T cell therapy in 2013 (3.5 years as reported in 2018) (22) after the patient received a T cell product with considerably higher EBV reactivity (38.46% of CD8^+^ T cells reacted to EBV) (21). Although participant 12 had received T cell therapy with very strong EBV reactivity (29.85% of CD8^+^ T cells reacted to EBV), the reactivity of the CD8^+^ T cells was directed at only EBNA1 (22).

*The year* 3 *assessment*

Participant 2 did not consent to the year 3 assessment. Three of the 6 participants who received T cell therapy with strong EBV reactivity (participants 3, 4, and 5) experienced an improvement in one or more of their MS symptoms at the year 3 assessment compared to baseline (Table 2). Energy levels and productivity were improved in participant 3, although there was an increase in left lower limb weakness. In participant 4, improvements in diplopia and lower limb spams, and resolution of longstanding vertigo, symptomatic lower limb clonus and neuropathic pain, which occurred at 6 months, were sustained at 3 years. Remarkably, all pain medications (paracetamol and amitriptyline) were ceased in this participant at 10 months as they were no longer required, and she remained pain-free at year 3 with an increase in QOL score (Figure 1F) and color vision at 3 years compared to baseline. However, her Fatigue Severity Scale score, which was reduced at year 2, increased to above the baseline level (Figure 1D) and there was further deterioration in left hand function due to weakness and ataxia. In participant 5 there were sustained improvements in productivity, manual dexterity, sleep quality, nocturia, urinary and fecal urgency, lower limb spasticity, distal lower limb power, coordination in the lower limbs, and lower limb sensation at year 3, with an associated reduction in the Fatigue Severity Scale score (Figure 1D) and an increase in QOL score (Figure 1F) compared to baseline. Despite these sustained improvements her walking deteriorated between year 2 and year 3 so that her EDSS score increased from 6.0 at year 2 to 6.5 (the baseline score) at year 3 (Figure 1B), although she was still able to walk 1,000 m with bilateral assistance, compared to 100 m at baseline. In participant 13 the EDSS score, which had been decreased at year 2 compared to baseline, increased from 4.5 to 6.5 at year 3 which was higher than her baseline score of 5.0 (Figure 1B). Of all the participants in the study, participant 5 received T cells with the highest EBV reactivity [45.45% of CD8^+^ T cells reacted to EBV (22)] and had the greatest degree of sustained clinical improvement at year 3.

At the year 3 assessment, 4 participants had the same EDSS score as at baseline (participants 1, 3, 4, and 5). All of these participants had shown clinical improvement at 6 months. Participant 1 had received a T cell product with weak EBV reactivity, and participants 3, 4, and 5 had received a T cell product with strong EBV reactivity. Five participants (participants 6, 8, 9, 12, and 13) had an increased EDSS score at year 3 compared to baseline (Figures 1A,B). Participants 6 and 8 had received a weak T cell product and had not improved at 6 months, whereas participants 9, 12, and 13 had received a strong T cell product and had shown clinical improvement at 6 months.

### Neuropsychological Assessment

Neuropsychological data are presented in Table 3. After correction for multiple comparisons there were no significant differences between the year 2 or 3 assessments and the baseline assessments for any of the 15 neuropsychological tests. The increase in verbal generativity that was observed on the Controlled Oral Word Association Test (COWAT) 6 months after T cell therapy (22) was maintained at both the year 2 and 3 assessments, whereas the increased attainment in the Paced Auditory Serial Addition Test (PASAT3), a measure of working memory/processing speed, observed at the 6-month assessment, (22) trended back toward baseline attainment at years 2 and 3. Depressive symptomatology was stable across the 3 year follow-up period, although participant 2 who experienced decreased mood at 6 months and 2 years after T cell therapy did not consent to the year 3 assessment, and participant 8 who also experienced decreased mood 6 months after T cell therapy did not consent to either the year 2 or the year 3 assessment.

**Table 3:** Comprehensive neuropsychological assessment data.

**Test**	**Baseline**	**Month 6**	**Year 2**	**Year 3**
COWAT	29.9 ± 6.6 (10)	35.0 ± 8.0 (10)	35.3 ± 9.3 (9)	34.6 ± 8.0 (7)
JOLO	25.3 ± 4.8 (10)	25.1 ± 4.8 (10)	24.6 ± 3.0 (9)	23.9 ± 4.5 (7)
CVLT-TL	47.1 ± 8.3 (10)	46.9 ± 9.7 (10)	48.1 ± 12.9 (9)	48.4 ± 12.6 (7)
CVLT-DR	8.4 ± 3.7 (10)	9.8 ± 3.3 (10)	10.9 ± 4.0 (9)	9.4 ± 5.0 (7)
BVMT-TL	21.7 ± 8.3 (10)	18.9 ± 7.2 (10)	22.5 ± 6.7 (8)	18.5 ± 5.9 (6)
BVMT-DR	7.9 ± 3.0 (10)	7.9 ± 2.6 (10)	8.8 ± 2.3 (8)	7.7 ± 2.7 (6)
SDMT	41.3 ± 12.3 (10)	41.0 ± 12.3 (10)	36.8 ± 13.7 (9)	35.9 ± 17.3 (7)
PASAT3	37.7 ± 13.8 (10)	41.4 ±12.8 (10)	38.3 ± 14.9 (9)	36.3 ±15.3 (7)
PASAT2	27.7 ± 11.3 (10)	31.4 ± 11.3 (10)	28.3 ± 12.7 (9)	25.7 ± 16.3 (7)
DKEFS-CS	8.1 ± 1.7 (10)	8.6 ± 2.0 (10)	8.7 ± 2.0 (9)	8.4 ± 3.6 (7)
DKEFS-DS	29.0 ± 6.4 (10)	30.1 ± 7.0 (10)	30.9 ± 8.4 (9)	27.7 ±12.4 (7)
Simple RT	0.40 ± 0.06 (9)	0.41 ± 0.05 (9)	0.43 ± 0.05 (7)	0.41 ± 0.06 (6)
Choice RT	0.71 ± 0.08 (9)	0.70 ± 0.07 (9)	0.73 ± 0.10 (8)	0.75 ± 0.07 (6)
Semantic RT	1.10 ± 0.22 (9)	1.03 ± 0.16 (9)	1.04 ± 0.17 (8)	1.00 ± 0.15 (6)
BDI	3.3 ± 2.7 (10)	3.1 ± 4.8 (10)	3.0 ± 5.1 (9)	1.7 ± 1.5 (7)

*Comprehensive neuropsychological testing was performed at baseline and then at 6 months, 2, and 3 years after EBV-specific T cell therapy. Mean ± standard deviation (number of participants assessed). For the first 11 parameters a higher score indicates better performance. In contrast, for the RT parameters a higher score represents a worse performance, and a higher BDI score represents more depressive symptoms. The results for the Simple, Choice and Semantic RT Tests in participant 6 were disregarded at all time points because of impaired manual dexterity. At years 2 and 3 participant 9 was unable to hold a pencil and therefore could not complete the BVMT-TL and BVMT-DR. The results for the Simple RT Test in participant 13 at year 2 were disregarded owing to an error in the administration of the test. COWAT, Controlled Oral Word Association Test; JOLO, Judgement of Line Orientation; CVLT-TL, California Verbal Learning Test—Total Learning; CVLT-DR, California Verbal Learning Test—Delayed Recall; BVMT-TL, Brief Visual Memory Test—Total Learning; BVMT-DR, Brief Visual Memory Test—Delayed Recall; PASAT3, Paced Auditory Serial Addition Test Inter-stimulus Interval 3 s; PASAT2, Paced Auditory Serial Addition Test Inter-stimulus Interval 2 s; DKEFS-CS, Delis Kaplan Executive Function System Sorting Test—Correct Sorts; DKEFS-DS, Delis Kaplan Executive Function System Sorting Test—Description Score; RT, Reaction Time; BDI, Beck Depression Inventory*.

### MRI Brain and Spinal Cord Scans

The MRI brain scan findings are presented in Table 4. Five participants (participants 1, 4, 6, 8, and 9) had one gadolinium-enhancing MRI brain lesion at baseline. In participant 6, the enhancing lesion did not change throughout the observation period and was thought to be unrelated to MS. Compared with the 6-month scan, the number of enhancing lesions at year 2 increased in 3 participants (participants 1, 2, and 9) and decreased in participant 4. Participants 1 and 2 experienced a clinical decline between the 6-month and 2-year assessments whereas participants 4 and 9 experienced some degree of sustained clinical improvement; therefore the change in number of enhancing brain lesions did not appear to be related to the clinical course. Participants 1, 2, 4, 6, 9, and 13 had 1–3 new lesions at year 2, compared to month 6, whereas participants 3, 5, and 12 had no new lesions. Participants 4 and 9 had one enhancing brain lesion at year 3. Participants 1, 4, 5, 9, and 13 had one new lesion at year 3 compared to year 2, whereas participants 6 and 12 had no new lesions at year 3.

**Table 4:** Brain MRI findings after T cell therapy.

**Participant Code**	**No. of enhancing T1 lesions at baseline**	**No. of enhancing T1 lesions at month 6**	**No. of new T2 lesions at month 6 compared with baseline**	**No. of enhancing T1 lesions at year 2**	**No. of new T2 lesions at year 2 compared with month 6**	**No. of enhancing T1 lesions at year 3**	**No. of new T2 lesions at year 3 compared with year 2**
1	1	0	1	1	1	0	1
2	0	0	0	1	2	NA	NA
3	0	0	0	0	0	NA	NA
4	1	2	4	1	3	1	1
5	0	0	0	0	0	0	1^b^
6	1^a^	1^a^	0	1^a^	I^b^	I^a^	0
8	1	0	0	NA	NA	NA	NA
9	1	1	4	2	2	1	1
12	0	0	2	0	0	0	0
13	0	0	1	0	1^b^	0	1

a *this lesion was unchanged throughout the observation period and thought to be unrelated to MS, most likely a capillary telangiectasia*.

b *indicates a spinal cord lesion; all other numbers in the table refer to lesions within the brain*.

*No., number; NA, result not available because MRI scans were not performed*.

### Intrathecal IgG Synthesis

CSF IgG production and leukocyte count were assessed in 8 participants at year 2, and 6 participants at year 3 (Table 5). Of the four participants with decreased intrathecal IgG synthesis 6 months after T cell therapy (participants 1, 6, 9, and 13), two of these (participants 6 and 13) still had decreased intrathecal IgG production at year 3 compared to baseline. In participant 13, ocrelizumab, which was commenced 2 months before the year 3 lumbar puncture, might have contributed to the decreased intrathecal IgG synthesis at year 3, but not at year 2 (Table 5). In participants 1 and 9, who had shown decreased intrathecal IgG synthesis 6 months after T cell therapy, the level of intrathecal IgG production had increased to above the baseline level by year 2. In participant 4, intrathecal IgG synthesis was increased at year 2, but decreased at year 3, compared to baseline.

**Table 5:** CSF IgG analysis and leukocyte count after T cell therapy.

**Participant code**	**Time of sample collection**	**CSF IgG Index (Reference range <0.70)**	**IgG (loc)^a^**	**IGGPROD (mg/L)^b^**	**Leukocyte count (**× 10**^6^/L)**
1	Baseline	0.69	1.6	7.8	1
	Month 6	0.61	−2.3	5.2	<1
	Year 2	0.93	12.2	18.5	2
	Year 3^c^	0.78	5.4	12.0	1
2	Baseline	0.90	20.9	32.8	2
	Month 6	0.88	19.1	31.3	4
	Year 2	0.96	22.9	33.6	3
	Year 3	Patient did not consent			
3	Baseline	0.60	−8.5	9.5	<1
	Month 6	Patient did not consent			
	Year 2	LP contraindicated owing to anti-coagulation			
	Year 3				
4	Baseline	0.89	17.6	28.6	1
	Month 6	1.04	25.8	35.2	8
	Year 2	1.32	29.6	36.8	<1
	Year 3	0.79	5.3	12.5	1
5	Baseline	0.48	−14.8	−2.5	<1
	Month 6	0.48	−13.1	−2.2	<1
	Year 2	0.47	−15.3	−3.1	<1
	Year 3	0.50	−10.7	−0.9	<1
6	Baseline	0.59	−5.5	5.7	<1
	Month 6	0.50	−15.0	−1.2	3
	Year 2	Blood-stained sample^d^	−11.7	−0.4	1
	Year 3	0.50			
8	Baseline	0.93	17.1	25.9	8
	Month 6	1.08	25.1	33.7	2
	Year 2	Patient did not consent			
	Year 3	Patient did not consent			
9	Baseline	0.68	2.1	13.2	<1
	Month 6	0.63	−23.8	−8.5	<1
	Year 2	0.79	8.7	17.7	<1
	Year 3	Blood-stained sample^d^			
12	Baseline	0.55	−9.2	3.3	2
	Month 6	0.57	−8.3	6.2	2
	Year 2	0.52	−12.6	0.6	1
	Year 3	Unsuccessful LP			
13	Baseline	1.03	19.4	26.7	2
	Month 6	0.70	5.7	15.7	3
	Year 2	0.88	12.1	19.4	4
	Year 3^e^	0.68	1.7	9.8	1

a * intrathecal IgG production (IgG(loc)) was calculated by the formula of Reiber and Felgenhauer: (30) IgG(loc) (mg/L) = [(CSF IgG ÷ serum IgG) – [0.8 × (√((CSF albumin ÷ serum albumin)2 + 15))] + 1.8] × serum IgG*.

b *intrathecal IgG production (IGGPROD) was calculated by the formula of now and colleagues: (31) CSF IgG-[(0.51 × CSF albumin × serum IgG)/serum albumin]*.

c *this patient received ocrelizumab 3 months prior to the year 3 CSF examination*.

d *the results of this sample were disregarded because the CSF was contaminated with proteins and cells from the blood*.

e *this patient commenced ocrelizumab 2 months prior to the year 3 CSF examination*.

*LP, lumbar puncture*.

## Discussion

In the current study we followed up ten patients with progressive MS for 2 years after autologous EBV-specific T cell therapy and nine of these patients for 3 years after T cell therapy. There were no serious treatment-related adverse events. Of the seven patients who had shown clinical improvement 6 months after T cell therapy (22), four still had at least some degree of sustained clinical improvement at year 2, with two of these still having a reduced EDSS score compared to baseline. At year 3, three of the participants who had clinical improvement at month 6 still showed some evidence of sustained clinical improvement, although the EDSS score that had still been reduced at year 2 had increased to baseline in one patient and above baseline in the other. We also observed a reduction in the Fatigue Severity Scale score compared to baseline in three of the participants with sustained clinical improvement at year 2, as we did in the participants with clinical improvement 6 months after T cell therapy (22). These clinical improvements lasting for up to 3 years after EBV-specific T cell therapy are contrary to the expected decline of patients with progressive forms of MS (32).

We have previously reported that clinical improvement after EBV-specific T cell therapy is associated with a higher level of EBV-specific CD8^+^ T cell reactivity in the administered T cell product (22). In the present study all four of the participants with some degree of sustained clinical improvement at year 2 and all three of the participants with some sustained improvement at year 3 had received T cells with strong EBV reactivity. Remarkably, participant 5 who had received T cells with the strongest EBV reactivity and who had shown pronounced neurological improvement and reduction in fatigue at 6 months had the greatest degree of sustained improvement in neurological function and fatigue 3 years after T cell therapy. These findings suggest that EBV-specific T cell therapy has a beneficial effect in progressive MS.

Previously we have postulated that clinical improvement following EBV-specific T cell therapy results from the killing of EBV-infected B cells in the CNS by the adoptively transferred CD8^+^ T cells and that this prevents further autoimmune attack on the CNS and allows neurological recovery through remyelination, dendritic and axonal sprouting, and synaptic remodeling (22). Similarly, this might also account for the sustained clinical improvements we observed in four participants at year 2 and three participants at year 3 after T cell therapy. An important goal of future studies should be to determine whether EBV-specific T cell therapy decreases the number of EBV-infected B cells in the CNS and whether this correlates with clinical improvement in MS. However, at present there is no safe and reliable way of quantifying EBV-infected B cells and plasma cells in the CNS.

A key question is why the clinical improvement in responders waned over time. With increasing duration of MS there is a progressive decrease in the frequency of EBV-specific T cells in the CD8^+^ population, indicating T cell exhaustion (33, 34). We suggest that CD8^+^ T cell exhaustion induced by a persistently high EBV latent antigen load in MS might gradually decrease the cytotoxic effect of the adoptively transferred CD8^+^ T cells on EBV-infected B cells in the CNS and thereby allow further autoimmune attack on the CNS. Repeated administration of EBV-specific T cell therapy every 12 months should help sustain clinical improvement in MS.

Limitations of our study include the small cohort of participants and the relatively high level of disability prior to treatment. These factors could impact upon the ability to detect clinical or radiological change over the follow-up period. The fact that some participants displayed clinical improvement is remarkable but should be interpreted with some caution. For example, participant 9 had an improved Fatigue Severity Scale score 2 and 3 years after T cell therapy when the EDSS score had increased. In this case, the reduction in reported fatigue might have been due to reduced energy expenditure as a result of decreased physical activity. Future studies incorporating activity monitoring devices could provide more insight.

This study adds to the accumulating evidence for a pathogenic role of EBV infection in MS. Our data suggests that T cell therapy targeting EBV-infected B cells in MS patients has a favorable safety profile and the potential for long-term efficacy. A phase I clinical trial of allogeneic EBV-specific T cell therapy is currently in progress (35).

## Data Availability Statement

The original contributions presented in the study are included in the article/supplementary material, further inquiries can be directed to the corresponding author/s.

## Ethics Statement

This follow-up study was approved by the Royal Brisbane and Women's Hospital Human Research Ethics Committee and The University of Queensland Medical Research Ethics Committee. All participants provided written informed consent. The patients/participants provided their written informed consent to participate in this study.

## Author Contributions

MP conceived and designed the research. ZI, PC, ND, GM, AS, KT, TH, KH, KW, AC, and MP collected and/or analyzed the data. ZI wrote the manuscript with critical input from all authors. All authors contributed to the article and approved the submitted version.

## Conflict of Interest

ZI: Received clinical trial support from Atara Biotherapeutics. MP: Received research grants from Atara Biotherapeutics and MS Queensland, and consultancy fees, clinical trial support, and travel support from Atara Biotherapeutics. The remaining authors declare that the research was conducted in the absence of any commercial or financial relationships that could be construed as a potential conflict of interest.
